# Impact of temperature and imported cases on the spread and control of dengue fever: Case study of 2019 dengue fever epidemic in Guangzhou and Jinghong cities, China

**DOI:** 10.1371/journal.pntd.0013472

**Published:** 2025-09-22

**Authors:** Yue Zhang, Xianghong Zhang, Kaifa Wang

**Affiliations:** Department of Mathematics and Statistics, Southwest University, Chongqing, China; International Atomic Energy Agency, AUSTRIA

## Abstract

Dengue fever is an acute mosquito-borne disease transmitted by Aedes mosquitoes. In this paper, dengue fever outbreaks in Guangzhou, Guangdong Province and Jinghong, Yunnan Province from July 15 to November 20, 2019 were studied to explore the effects of temperature differences and imported cases on epidemic development patterns. In response to the practical issue of missing mosquito vector data, the feasibility of using meteorological data-driven dynamic model to obtain mosquito vector data was initially validated. Cross-correlation analysis was then used to assess the strong correlation between mosquito vector data and dengue cases. The relationship between bite rate, transmission rate, incubation period, mortality rate and effective reproduction number with respect to daily mean temperature (DMT) and daily temperature difference (DTR) was established by maximum likelihood estimation. The results of sensitivity analysis showed that the most sensitive parameters to basic reproduction number were mosquito mortality and transmission rate of dengue virus between mosquito vectors and humans. The results of comparative analysis showed that the temperature difference between Guangzhou and Jinghong was the main factor contributing to the difference of dengue epidemics in the two cities, because temperature could affect the development of dengue epidemics by affecting the living habits of mosquito vectors. In addition, imported cases and the intensity of epidemic prevention measures are also important factors leading to the difference in dengue epidemics between the two places. Therefore, the key to the prevention and control of dengue fever is to implement mosquito elimination as soon as possible according to the change of temperature, raise public awareness of mosquito prevention and epidemic prevention, and strengthen the control of imported cases.

## Author summary

The rapid development of dengue fever in China has become a public health problem that cannot be ignored. However, the situation of each epidemic area is different, so these differences need to be analyzed and studied separately to find more effective directions for dengue outbreak management and control. Studies have shown that climate and imported cases are important factors affecting local dengue cases in China. To verify the influence of these two factors, we chose Guangzhou and Jinghong, two regions with certain differences in geographical location and climate. In this study, we found that the influence of temperature on mosquito transmission behavior in Guangzhou and Jinghong is different to some extent due to climate differences. At the same time, due to the geographical location of the two regions, the impact of imported cases on the number of local cases is also different. These results suggest that future epidemic prevention strategies need to be adapted to local conditions, adapting public health strategies to the climate, geography and environment of each region to achieve effective prevention and control.

## 1. Introduction

Dengue is one of the most common mosquito-borne infectious diseases transmitted by Aedes mosquitoes (primarily Aedes albopictus and Aedes aegypti) and is mainly prevalent in tropical and subtropical regions. In recent years, with global warming, the epidemic of dengue fever has become more severe, and cases have been reported in more than 100 countries and regions [1]. The southern regions of the Chinese mainland, including Guangdong, Guangxi, Hainan, Zhejiang, Fujian and Yunnan provinces, have the highest incidence of dengue fever. Dengue cases have been reported almost every year in Guangdong and Yunnan provinces since the 2000s.

Dengue transmission is strongly influenced by meteorological factors, particularly temperature, which affect mosquito behavior and virus transmission dynamics [2,3]. While most studies use monthly mean temperatures or constant-temperature experimental data [4–6], however, in nature, daily temperature fluctuations have an important impact on mosquitoes and their pathogens. The importance of considering the change of daily mean temperature (DMT) and daily temperature variation (DTR) when studying the transmission dynamics of dengue virus has been proposed [7]. In 2014, Guangdong Province experienced the worst outbreak of dengue fever in China to date. To identify the determinants of the outbreak in Guangzhou in 2014, statistical methods such as cross-correlation analysis and likelihood estimation were used to quantified the effects of vector parameters, DMT and DTR on the dengue epidemic, and to verify the key role of mosquito density and daily mean temperature in the transmission of dengue fever in Guangzhou [8]. From the perspective of dynamic modeling, a deterministic infectious disease model was established considering climate factors, imported cases, vertical transmission and local intervention measures. The results showed that imported dengue cases, mosquito density and weather variables played a key role in the transmission of dengue fever [9]. Controlling local transmission of dengue fever is also a major concern for many researchers [10,11]. In fact, global warming, widespread vector-borne transmission, frequent population movements, and inappropriate control strategies all increase the risk of dengue transmission and pose a serious threat to public health security.

In 2019, China experienced a major outbreak of dengue fever, with Guangdong and Yunnan provinces particularly affected [12,13]. According to the China Public Health Science Data Center [14], the cumulative number of cases in China in 2019 was 22,188, Yunnan Province had the highest number of cases (6,471), followed by Guangdong Province (6,042), with Guangzhou and Jinghong respectively being the main centers of dengue outbreaks in these two provinces. Although Guangzhou and Jinghong were the cities with large outbreaks of dengue fever in 2019, there were significant differences in climate, foreign import intensity, geographical location and population density between the two regions. Therefore, this paper selected these two cities as comparative studies to explore the impact of temperature and imported cases on the transmission of dengue fever in the two cities, and to reveal the underlying mechanisms.

Noted that there are also differences in the main vectors for dengue fever transmission between the two places. In Guangzhou, the primary vector for dengue transmission is Aedes albopictus, whereas in Jinghong, both Aedes albopictus and Aedes aegypti serve as the major vectors [15]. Since there are similar temperature-dependent mosquito vector parameters for the two mosquito species, such as daily bite rate, transmission probability, external latency period, mortality rate and so on [3,7,13], for simplicity, based on a unified Aedes mosquito ecological model framework, we achieved regional adaptive adjustment of the model through using local temperature data and dengue cases to dynamically calibrate the model parameters, thereby ensuring the applicability of the model in different epidemiological scenarios. This processing method not only maintains the universality of the model structure but also considers regional specificity.

To address the lack of mosquito surveillance data, this study reconstructed vector population dynamics using a mechanistic model [16], with its reliability validated by Pearson correlation analysis between model simulated mosquito data and mosquito surveillance data. Cross-correlation analysis was further employed to quantify the spatiotemporal relationship between mosquito abundance and dengue cases. Through maximum likelihood estimation, we established quantitative associations between critical parameters (biting rate, transmission rate, etc.) and daily mean temperature (DMT) as well as daily temperature variation (DTR). Sensitivity analysis was subsequently conducted to identify the most influential parameters on the basic reproduction number. This study features three innovations: 1) Comparative analysis between Guangzhou and Jinghong to uncover differential impacts of temperature, imported cases, and control measures; 2) Temperature-driven dynamic modeling to reconstruct vector populations, addressing the gap in traditional mosquito surveillance data; 3) Integrated statistical-dynamic modeling framework to elucidate multifactorial transmission mechanisms for targeted prevention.

The rest of this paper is structured as follows: Section 2 provides a brief description of the data, models and statistical methods used. In Section 3, the model-driven mosquito vector data and Pearson correlation analysis results are presented. Cross-correlation analysis is used to explore the time-delayed effect of the change in the number of mosquito vectors on the daily number of new cases, the maximum likelihood estimation method is used to evaluate the vector parameters, and the change trend of the effective reproduction number in the two places is compared to analyze the possible factors causing the different development trends of the epidemic in the two places. The influence of temperature on the development of epidemic was verified by sensitivity analysis. Section 4 discusses the important factors contributing to the differences in dengue epidemics between Guangzhou and Jinghong and proposes that control of mosquitoes and imported cases contributes to disease control.

## 2. Materials and methods

### 2.1. Study area

From July 15 to November 20, 2019, there are 1,174 reported cases in Guangzhou, while 3,206 cases were reported in Jinghong [14]. Guangzhou is the capital city of Guangdong Province, located in south China. It has a subtropical monsoon climate. It is hot and humid in summer and has always been an important port city in China’s foreign trade. Jinghong is in the southern part of Yunnan Province, with a tropical and subtropical humid monsoon climate, bordering Myanmar to the south, Laos and Thailand. According to meteorological data, from July 15 to November 20, 2019, the average daily temperature in Guangzhou ranged from 19 to 34°C, while that in Jinghong City ranged from 9.5 to 23°C [17]. In addition, the population density of Guangzhou is 2,512 per/km², much higher than Jinghong City’s 78per/km² [18,19]. As an important trade center, Guangzhou has a large floating population, while Jinghong City has a significant population movement due to its proximity to the border, especially in the context of serious dengue epidemics in Southeast Asian countries such as Myanmar. The aim of this study was to investigate the effects of temperature difference and import intensity on the transmission of dengue fever in Guangzhou and Jinghong.

### 2.2. Data collection

#### 2.2.1. Dengue data.

**Fig 1:**
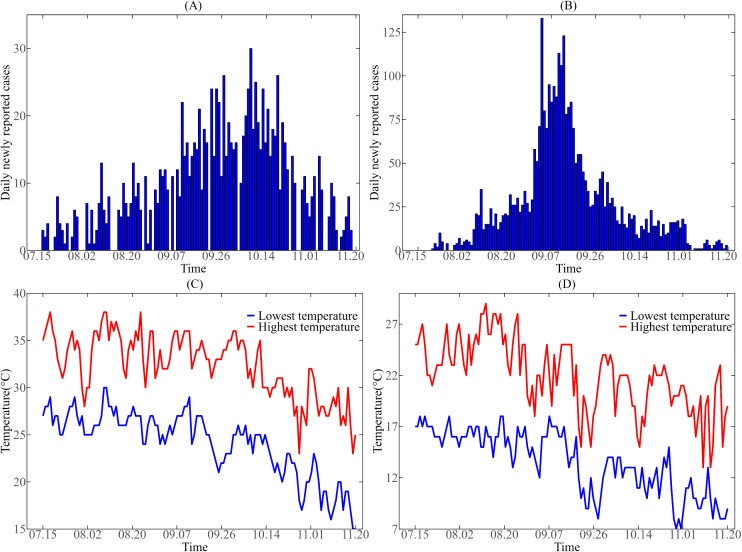
Daily new dengue cases and temperature data from July 15 to November 20, 2019. **(A)** Daily new dengue cases in Guangzhou; **(B)** Daily new dengue cases in Jinghong; **(C)** Temperature data in Guangzhou; **(D)** Temperature data in Jinghong.

Since the daily new dengue case data of Guangzhou and Jinghong in 2019 could not be found on the public health data disclosure platform of these two cities, we referred to the dengue case data of these two cities in 2019 from previous studies [16,20]. The data in these literatures were obtained from the CDC surveillance system of the two cities. Fig 1(A, B) show the daily data of new cases in Guangzhou and Jinghong from July 15 to November 20, 2019. Detailed daily data of new cases can see S2 File.

#### 2.2.2. Meteorological data.

To investigate the effects of daily mean temperature DMT and daily temperature variation DTR on dengue transmission, we obtained the daily record maximum and minimum temperatures in Guangzhou and Jinghong during July 15 to November 20, 2019, from the Data Center of China Meteorological Administration, and calculated the DMT and DTR of the two cities accordingly (see S2 File). In addition, we assume that the daily temperature changes in a sinusoidal curve between the highest and lowest values, dividing the day into 48 periods to calculate the temperature at any time of the day. The daily maximum temperature (red line) and daily minimum temperature (blue line) in Guangzhou are shown in Fig 1(C), while the daily maximum and minimum temperatures in Jinghong are shown in Fig 1(D).

#### 2.2.3. Mosquito vector data.

**Fig 2:**
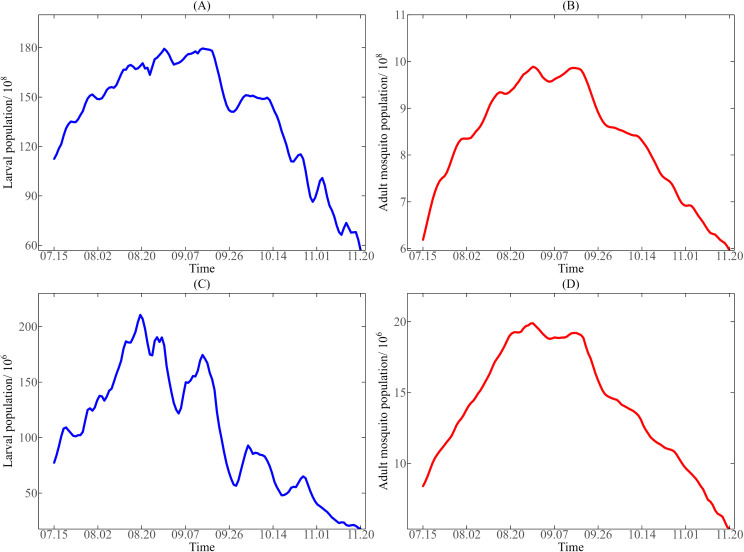
The number of aquatic-stage larvae and adult mosquitoes obtained by the dynamic model from July 15 to November 20, 2019. **(A-B)** The number of aquatic-stage larvae and adult mosquitoes in Guangzhou; **(C-D)** The number of aquatic-stage larvae and adult mosquitoes in Jinghong.

***Model-driven mosquito vector data.*** The mosquito vector detection data of Guangzhou and Jinghong in 2019 was not found on the public health data disclosure platforms of these two cities, so we will consider using the existing dynamic model to estimate the mosquito vector data during the epidemic period in Guangzhou and Jinghong in 2019. SEIR model is a commonly used infectious disease dynamic model, which is mainly used to describe the transmission process of infectious disease in the population. In this paper, based on the coupled transmission mechanism of dengue fever, a dengue transmission model (ELPSEI-SEIR) [16] was further established based on SEIR model and other relevant studies [2]. The Markov Chain Monte Carlo (MCMC) algorithm was used to fit the daily reported cases of dengue fever in Guangzhou and Jinghong with the dengue transmission model, and the estimates of unknown model parameters in the two cities were obtained respectively, so as to obtain the simulated number of aquatic-stage larvae and adult mosquitoes (see S2 File). They were used as the simulated values of mosquito vector data used in this study. The model-driven mosquito vector data of Guangzhou and Jinghong are shown in Fig 2. The detailed dynamic model description and parameter estimation results are shown in S1 File.

***Mosquito surveillance date****.* We also collected the weekly mosquito vector density surveillance date of Panyu District, Guangzhou from July 15 to November 20, 2019, from the official platform of the Guangzhou Center for Disease Control and Prevention, including Breteau Index (BI) and Mosquito Ovitrap Index (MOI) (see S2 File), as the actual mosquito vector data for subsequent correlation analysis [21]. Breteau Index (BI) refers to the number of containers of Aedes mosquito larvae in every 100 households, which is a key indicator to evaluate the density of Aedes mosquitoes in an area. Mosquito Ovitrap Index (MOI) is a key entomological surveillance metric for adult mosquito density surveillance. It represents the percentage of ovitraps that are positive for Aedes eggs, larvae, or adult mosquitoes out of the total number of effectively collected traps. Positive ovitrap means it contains at least one Aedes egg, larva, or adult. BI and MOI are often used by the public sector to monitor and assess the risk of transmission of mosquito-borne infectious diseases.

### 2.3. Correlation analysis

In the public data open platform of Guangdong Province and Yunnan Province Center for Disease Control and Prevention, we failed to find the daily BI and MOI in Guangzhou and Jinghong in 2019, only found the relevant weekly data in Panyu District of Guangzhou. The correlation between the estimated data in Guangzhou and the real data in Panyu District was used to confirm the feasibility of the data estimation method.

Considering that Panyu is a district of Guangzhou and the main outbreak area of dengue fever, there is a certain correlation between the actual number of mosquito vectors in Guangzhou and Panyu District. Therefore, we compiled the simulated values for the number of aquatic-stage larvae and adult mosquitoes in Guangzhou during the period from July 15 to November 20, 2019, respectively, into weekly data. Subsequently, Pearson correlation analysis was conducted between the model-driven mosquito vector data and the actual weekly mosquito vector surveillance data in Panyu District by using R language software. The results showed that the model-driven data of mosquito vectors in Guangzhou in 2019 were significantly correlated with the actual surveillance data in Panyu District. In addition, considering that there may be a time lag between the number of mosquito vectors and the number of new daily cases, we used cross-correlation analysis to examine the interaction between the number of mosquito vectors and the number of new daily cases over time. Cross-correlation analysis is a spectral analysis method for analyzing time series of two random variables [22,23], which can be used to detect the association between mosquito vector data and the daily number of new cases.

### 2.4. Maximum likelihood estimation

Vector capacity is a key indicator of vector epidemiological potential. To study the effect of temperature on disease transmission, it is necessary to determine the specific expression of each vector parameter with respect to temperature.

#### 2.4.1. Vector capacity.

The basic reproduction number $\;(R_{0}\backslash )$represents the average number of new cases arising during the infection period $\;(T_{h}$) after a typical infected person is introduced into a fully susceptible population. The effective reproduction number ($R_{\text{0}}(t)$) represents the average number of secondary cases produced by a typical infected individual at time point t in a population that is not entirely susceptible (due to immunity, interventions, or other factors). Vector capacity $\left(V_{c}\right)$ is one of the important indicators describe vector communication ability, on behalf of the daily amount of reproduction [3,24]. Relative vector capacity ($R_{vc}$) [25] refers to the ratio of vector capacity to the population, and a higher relative vector capacity indicates a higher likelihood of dengue epidemics. The relative vector capacity $\left(R_{vc}\right)$ can be expressed as


$$R_{vc}=\frac{a^{2}b_{h}b_{m}\text{exp}\left(-\mu_{m}n\right)}{\mu_{m}},$$ (1)

where a is the daily average vector bite rate. $b_{h}$ represents the probability that a mosquito will infect a person after each bite. $b_{m}$ is the probability of transmission from person to mosquito per bite. n is the duration of external latency period (EIP). $\mu_{m}$ is the vector mortality rate. The specific relationship between $V_{c}$, $R_{vc}$ and $R_{0}$ is as follows [3,24,25]:


$$V_{c}=\frac{R_{0}}{T_{h}},$$

$$R_{vc}=\frac{V_{c}}{m}=\frac{R_{0}}{T_{h}m},$$

$$R_{0}=R_{vc}T_{h}m,$$

where m is the proportion of the mosquito population to the human population.

#### 2.4.2. Temperature and mosquito vector parameters.

Since the five mosquito-borne vector parameters ($a,\;b_{h},\;b_{m},\;n$ and $\mu_{m}$) depend on temperature, $R_{vc}$ and $R_{0}\;$will also be affected by temperature changes. We need to further estimate the daily $R_{vc}$ and $R_{0}$ by estimating the above five critical parameters. First, let x=DMT,y=DTR, and assume that the temperature of a day is sinusoidal between (x±y/2). We then divide the day into 48 equal periods and denote the temperature at the time point $t_{i}$ as $\;T_{t_{i}}$. The values of the above five vector parameters at each time point are recorded as $a^{\prime }$, $b_{h}^{\prime },\;b_{m}^{\prime },\;n^{\prime }$ and <Eqn33>>$\mu_{m}^{\prime },$ respectively. Note that the expression of the five vector parameters about temperature $T_{t_{i}}$ can be referred to [3,7,13,26,27], and the specific expression is as follows:


$$\left\{ \begin{array}{c} \begin{array}{c} a^{\prime }\left(T_{t_{i}}\right)=a_{1}T_{t_{i}}+a_{2},\;\text{12}.\text{4}\leq T_{t_{i}}\leq 32,\\ b_{h}^{\prime }\left(T_{t_{i}}\right)=a_{3}\left(T_{t_{i}}\right)\left(T_{t_{i}}-a_{4}\right)\sqrt{a_{5}-T_{t_{i}}},\text{12}.\text{286}\leq T_{t_{i}}\leq 32.461,\\ b_{m}^{\prime }\left(T_{t_{i}}\right)=\left\{ \begin{array}{c} a_{11}\left(T_{t_{i}}\right)-a_{12},12.4\leq T_{t_{i}}\leq 26.1,\\ 1,\;26.1<T_{t_{i}}\leq 32.5, \end{array}\right.\; \end{array}\\ n^{\prime }\left(T_{t_{i}}\right)=a_{13}+exp\left(a_{14}-a_{15}T_{t_{i}}\right),\\ \mu_{m}^{\prime }\left(T_{t_{i}}\right)=a_{6}+a_{7}T_{t_{i}}+a_{8}T_{t_{i}}^{2}+a_{9}T_{t_{i}}^{3}+a_{10}T_{t_{i}}^{4}, \end{array}\right.\;$$

where


$$T_{t_{i}}=\frac{y}{2}\text{sin}\left(\frac{t_{i}-6}{12}\pi \right)+x,\;t_{i}=0.5,1.0,1.5,\ldots ,\text{24},\;i=\text{1},\text{2},\ldots ,\text{48}.$$

By the above formula, we can obtain the values $a^{\prime },\;b_{h}^{\prime },\;b_{m}^{\prime },\;n^{\prime }$ and $\mu_{m}^{\prime }$ at 48 time points, and then their average values were taken as the daily bite rate, the probability of vector transmission to humans, the probability of vector infection, the length of vector incubation period and the mortality of the vector$\;(a,\;b_{h},\;b_{m},\;n,\;\mu_{m}\;\text{and}\;m)$. The specific expressions are as follows:


λ=1,2,4 (2)

Substituting the expression of the above vector parameters into Eq. (1), we can obtain


$$R_{vc}(x,y)=\frac{a^{2}(x,y)b_{h}(x,y)b_{m}(x,y)\text{exp}\left(-\mu_{m}(x,y)n(x,y)\right)}{\mu_{m}(x,y)}.$$ (3)

#### 2.4.3. Likelihood function and parameter estimations.

To estimate ${\;V}_{c},$ $R_{vc}\;\text{and}\;R_{0}$, we will use the likelihood-based method and the generation interval method. Assuming that the number of new cases per day follows the Poisson distribution [28], the likelihood function is as follows


$$likfuction=\prod_{t=1}^{D}\frac{\text{exp}(-\varphi_{t})\varphi_{t}^{N_{t}}}{\Gamma (N_{t}+1)},\;$$

where Γ is the gamma function. D is the number of days, $\;N=\{N_{1},...,N_{D}\},$ $N_{t}$ represents the number of new cases on day t, and


$$\varphi_{t}=R_{0}(t)\sum_{j=1}^{\text{min}(t,k)}p_{j}N_{t-j},$$ (4)

$$R_{0}(t)=R_{vc}(t)T_{h}m(t),$$ (5)

$$R_{vc}(t)=\frac{a^{2}(t)b_{h}(t)b_{m}(t)\text{exp}\left(-\mu_{m}(t)n(t)\right)}{\mu_{m}(t)},$$ (6)

$$m(t)=\frac{a_{16}M(t)}{N_{h}}.$$ (7)

Here the parameter k refers to the maximum value of the serial interval (4–10 days for both mosquito and human incubation periods) [1]. Let the generation interval j follows a gamma distribution with mean of 14 and a variance of 2, where $p_{j}$ represents its probability density function [8,28]. M(t) represents the number of adult mosquito vectors at time t obtained by the dynamic model (see S1 File).$\;N_{h}$ represents the human population of the study area. Some of the parameter definitions in the Eqs. (1)–(7) above are summarized in Table 1.

**Table 1:** The definition of parameters contained in Eqs. (1)-(7).

Parameters	Definition
a	Average daily bite rate of vector (human/mosquito/day)
m	The ratio of the number of mosquitoes to the population (mosquito/person)
ma	Average number of mosquito bites per person per day (count/ person/day)
$b_{h}$	Transmission probability from vectors to humans per bite (N/A)
$b_{m}$	Transmission probability from humans to vectors per bite (N/A)
n	Duration of EIP (days)
$\mu_{m}$	Vector mortality (N/A)
$T_{h}$	Infection period (days)
$a_{16}$	The proportion of female adult mosquitoes in an area
M(t)	The total number of adult mosquitoes on day t (N/A)
$N_{h}$	The total population of the area (person/${km}^{2}$)
$p_{j}$	The probability of generation interval for j days (N/A)
k	The maximum value of the serial interval (days)

In 2019, the total population of Guangzhou is 15,305,900, and the total population of Jinghong is 642,737 [18,19]. Due to the large number of unknown parameters, on the one hand, during the dengue epidemic in 2019, Jinghong and Guangzhou have adopted a series of control measures, and human intervention is the main factor affecting the mosquito-borne mortality. Therefore, we will ignore the influence of temperature on the mosquito-borne natural mortality $\mu_{m}$, set $a_{7}=a_{8}=a_{9}=a_{10}=0$. On the other hand, we refer to relevant literature and reasonable assumptions to fix the values of some parameters, as shown in Table 3 [1,3,29]. Based on the given likelihood function, combined with the daily number of new dengue cases in Guangzhou and Jinghong, we use the maximum likelihood estimation method to find the parameters that can maximize the likelihood function in the parameter space, so as to obtain the estimates of the remaining eight unknown parameters $a_{1},\;a_{2},\;a_{6},\;a_{11},\;a_{12},\;a_{13},\;a_{14}$ and $a_{15}$. Then, according to the relationship between the five vector parameters ($a,\;b_{h},\;b_{m},\;n\;\text{and}{\;\mu }_{m}$) and the estimated parameters, the DMT and DTR expressions of the vector parameters in the two regions can be derived. The setting and estimation results of related parameter values are shown in **Table 3**.

### 2.5. Sensitivity analysis

To assess the impact of six vector parameters ($a,b_{h},b_{m},\mu_{m},n,m$) on dengue transmission, we will analyze their sensitivity to the basic reproduction number $R_{0}$. The input parameters are sampled in their parameter space using Latin Hypercube Sampling (LHS). Sensitivity analysis was performed by calculating the Partial Rank Correlation Coefficient (PRCC) between the input parameters and the basic reproduction number to assess the impact of parameters on $R_{0}$ and dengue transmission [30–32]. In general, when the absolute value of the correlation coefficient (PRCC) is less than 0.2, the correlation is weak. When its absolute value is between 0.2 and 0.4, it indicates a certain degree of correlation. When the absolute value is greater than 0.4, the correlation is stronger. In addition, if PRCC is positive, it indicates a positive correlation, a negative value indicates a negative correlation.

Firstly, without considering the influence of temperature (DMT and DTR) on the vector parameters, parameter sensitivity analysis was performed through systematic sampling within predetermined ranges of input parameters ($\text{here}\;a,b_{h},b_{m},\mu_{m},n,m$) to quantify each parameter’s contribution to $R_{0}$ [3,27,33–35]. Secondly, considering that some vector parameters ($\text{here}\;a,b_{h},b_{m},n$) are closely related to DMT and DTR, we used LHS method to sample the values of DMT and DTR, while vector mortality ($\mu_{m}$) and the proportion of the mosquito population to the human population (m) within their ranges of the two study regions respectively. Then we take sample values of DMT, DTR, $\mu_{m}\;\text{and}\;m\;(\text{temperature}-\text{independent})$ as input parameters, calculate the values of mosquito vector parameters ($\text{here}\;a,b_{m},b_{h},n$) using their temperature-dependent expressions (Eq. (2)). Finally, the sensitivity of the vector parameters to the basic reproduction number under the influence of temperature was investigated by evaluating the PRCCs of the six parameters.

## 3. Results

### 3.1. Mosquito population

The model-driven data about the number of aquatic-stage larvae and adult mosquitoes in Guangzhou from July 15, 2019 to November 20, 2019, obtained by the dynamic model, are shown in Fig 2(A) and (2B). Similarly, Fig 2(C) and (2D) show the model-driven data about the number of aquatic-stage larvae and adult mosquitoes in Jinghong. As shown in Fig 2, the overall change of mosquito population in the two places showed a trend of first increasing and then decreasing. Specifically, the number of aquatic-stage larvae and adult mosquitoes in Guangzhou reached their peak around September 1, while the number of aquatic-stage larvae and adult mosquitoes in Jinghong reached their peak around August 20. Besides, the variation trend of adult mosquito population in Guangzhou and Jinghong was consistent, while the variation of larval population was different to some extent, and the fluctuation range of larval population in Jinghong was more severe.

### 3.2. Correlation analysis

The mosquito population (including aquatic-stage larvae and adult mosquitoes) in Guangzhou from July 15 to November 20, 2019, obtained by dynamic model simulation, were sorted into weekly data. The Pearson correlation analysis results between the actual weekly surveillance mosquito vector data (including BI and MOI) and model-driven weekly data in Panyu District of Guangzhou are shown in **Table 2**. Here, BI and MOI correspond to the actual mosquito vector density surveillance data in Panyu district, where BI represents the larval density data and MOI represents the adult mosquito density data.

**Table 2:** Pearson correlation analysis of weekly mosquito vector data simulated by dynamic model in Guangzhou and actual weekly mosquito vector data in Panyu District in 2019.

Mosquito vector variable	Simulated number of adult mosquitoes (/${\text{10}}^{\text{8}}$)	Simulated number of aquatic-stage larvae (/${\text{10}}^{\text{8}}$)
BI	0.5638(p = 0.0229)	0.5775(p=0.0192)
MOI	0.3928(p = 0.1323)	0.4581(p = 0.0743)

The results showed that there was a significant correlation between the mosquito population in Guangzhou and the BI weekly data in Panyu District (r = 0.5638, p < 0.05; r= 0.5775, p < 0.05). In addition, although the correlation analysis results between the mosquito number obtained by the model and the weekly MOI in Panyu District showed that the p-value was not lower than 0.05, there was still a certain degree of correlation between the two (r = 0.3928, p = 0.1323; r = 0.4581, p = 0.0743) in terms of experience [36]. These results confirmed the rationality of using dynamic models to obtain mosquito data.

**Fig 3:**
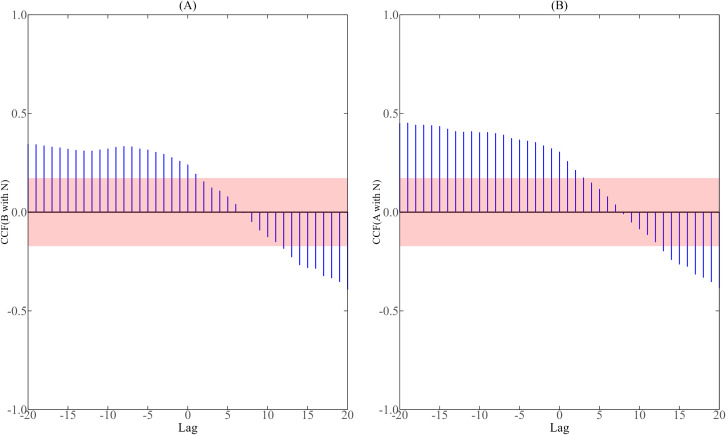
Cross-correlation analysis between the number of mosquito vectors and the daily reported cases in Guangzhou from July 15 to November 20, 2019.

**Fig 4:**
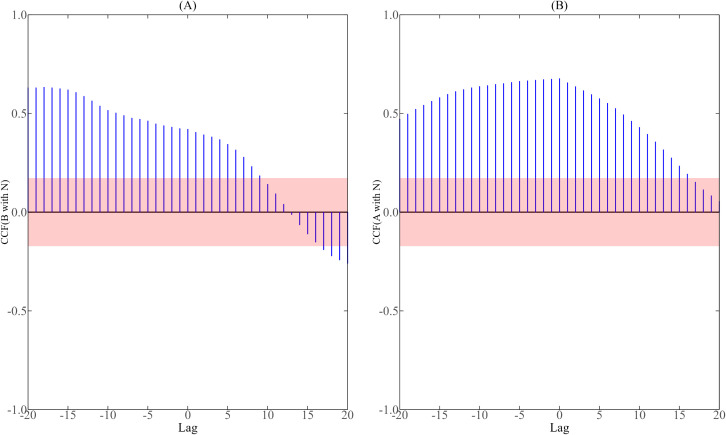
Cross-correlation analysis between the number of mosquito vectors and the daily reported cases in Jinghong from July 15 to November 20, 2019.

We further analyzed the correlation between the number of mosquito vectors and the number of new reported cases per day. During the dengue fever epidemic in 2019, the correlation analysis results of simulated mosquito vector data (i.e., the number of aquatic-stage larvae and adult mosquitoes) with the number of new cases in Guangzhou and Jinghong are shown in Fig 3 and Fig 4, and the red shadow in the figure is the 95% confidence interval of correlation coefficient. It is not difficult to find from Fig 3 and Fig 4 that the correlation coefficient between any two variables presents a continuous trend of change over time, because the number of aquatic-stage larvae and the number of adult mosquitoes are calculated by combining the continuous dynamic model of the number of new cases per day. As can be seen from Fig 3, in Guangzhou, the lag time between aquatic-stage larval abundance and daily new cases ranged from -20–1 day, while between adult mosquito populations and daily new cases it ranged from -20–3 days. Fig 4 demonstrates that in Jinghong, the lag time between mosquito vector abundance and daily new cases ranged from -20–9 days for aquatic-stage larvae and -20–16 days for adult mosquitoes, respectively. In combination with Fig 3 and Fig 4, it can be seen that there is indeed a significant correlation between the number of mosquito vectors and the number of new cases per day, and there is a certain time lag effect, and the delayed effect of changes in mosquito numbers on the number of new cases per day can reach 20 days at the longest. The results of cross-correlation analysis showed that the number of aquatic-stage larvae, the number of adult mosquitoes and the number of new cases per day had a feedback relationship and a concurrent relationship. This is similar to the conclusions obtained in previous related studies using BI and MOI as mosquito vector data [8].

### 3.3. Parameter estimation

#### 3.3.1. Maximum likelihood estimation.

Based on the constructed likelihood function, the maximum likelihood function estimation (MLE) is used to obtain the values of the unknown parameters and the corresponding 95% confidence intervals (CI). The parameter estimation results for Guangzhou and Jinghong are shown in **Table 3**.

**Table 3:** The fixed parameter, estimated parameters (in Eqs. (1)–(7)) and their 95% CI for Guangzhou and Jinghong cities are presented.

Parameter	Guangzhou	Jinghong	Reference
$a_{3}$	0.001044	0.001044	[3,7]
$a_{4}$	12.286	12.286	[3,7]
$a_{5}$	32.461	32.461	[3,7]
$a_{i},i=7,8,9,10$	0	0	[8]
*k*	20	20	[1]
$T_{h}$	5	5	[29]
$a_{1}$	0.0107(95%CI, 0.0103-0.0112)	0.0158(95%CI, 0.0146-0.0170)	Estimation
$a_{2}$	0.0103(95%CI, 0.0098-0.0107)	0.0893(95%CI, 0.0891-0.0894)	Estimation
$a_{6}$	0.3202(95%CI, 0.3177-0.3226)	0.3746(95%CI, 0.3745-0.3747)	Estimation
$a_{11}$	0.0623(95%CI, 0.0622-0.0624)	0.0231(95%CI, 0.0212-0.0249)	Estimation
$a_{12}$	0.6516(95%CI, 0.6515-0.6518)	0.1737(95%CI, 0.1735-0.1738)	Estimation
$a_{13}$	3.6289(95%CI, 3.6288-3.6290)	3.4868(95%CI, 3.4867-3.4870)	Estimation
$a_{14}$	5.3245(95%CI, 5.3244-5.3249)	5.2615(95%CI, 5.2610-5.2616)	Estimation
$a_{15}$	0.2200(95%CI, 0.2183-0.2217)	0.9986(95%CI, 0.9985-0.9990)	Estimation
$a_{16}$	0.0857(95%CI, 0.0819-0.0896)	0.5712(95%CI, 0.5711-0.5713)	Estimation

#### 3.3.2. Effect of temperature on mosquito vector parameters.

To visually demonstrate the effects of DMT and DTR on vector parameters, we drew contour maps to analyze the changes of these vector parameters under different DMT and DTR conditions. According to the temperature changes during the epidemic period in 2019, this paper investigated the effects of DMT ranging from 19°C to 34°C and DTR ranging from 3°C to 12°C on parameters in Guangzhou. Aiming at Jinghong, mainly analyzing the DMT in 11 °C to 25 °C and DTR between 0 °C to 15 °C impact on parameters.

**Fig 5:**
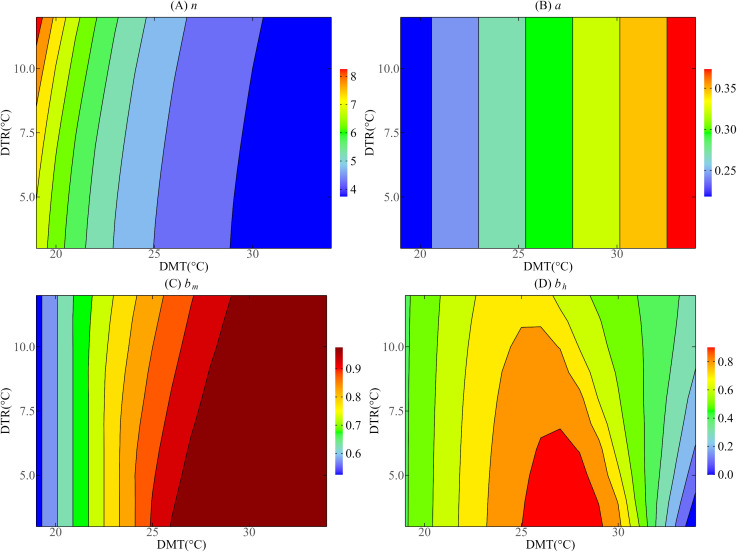
Contour plots of mosquito vector parameters with respect to DMT and DTR in Guangzhou.

**Fig 6:**
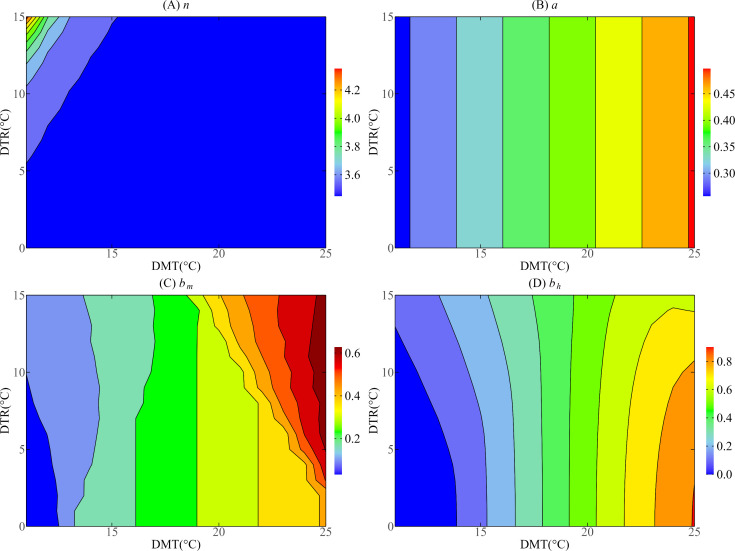
Contour plots of mosquito vector parameters with respect to DMT and DTR in Jinghong.

According to Fig 5(B) and Fig 6(B), there is a linear relationship between DMT and bite rate a. The greater the DMT, the greater the a is. However, the effects of DMT and DTR on other mosquito vector parameters showed nonlinear characteristics.

It can be seen from Fig 5(A) that the external incubation period (n) of mosquito vectors in Guangzhou decreased with the increase of DMT and increased with the increase of DTR. In contrast, Fig 6(A) shows that the length of mosquito vector external incubation period (n) in Jinghong decreases with the increase of DMT; when DMT is in the range of 0–16 °C, n increases with the increase of DTR, but when DMT > 16°C, n basically stays below 3.5 and is almost not affected by DTR.

As shown in Fig 5(C), in Guangzhou, when the DTR value is fixed, the probability of infection ($b_{m}$) per bite of mosquito vectors increases with the increase of DMT. When DMT ranges from 19 °C to 22°C, $b_{m}$ is not affected by the change of DTR, but when DMT ranges from 22°C to 29°C, $b_{m}$ decreases with the increase of DTR. When DMT > 29°C, $b_{m}$ is basically not affected by the change of DTR and remains at a high level. In contrast, Fig 6(C) shows that in Jinghong, when DTR = 0°C, $b_{m}$ increases with the increase of DMT, when DMT is in the range of 11–13°C and 19–25°C, $b_{m}$ increases with the increase of DTR, but when DMT is 13–19°C, $b_{m}$ decreases with the increase of DTR.

Fig 5(D) shows that in Guangzhou, when DTR = 0°C and DMT < 29°C, $b_{h}$ increases with the increase of DMT; when DMT > 29°C, $b_{h}$ begins to decline; when DMT = 20°C, $b_{h}$ is almost unaffected by DTR; when DMT is 22–32 °C, $b_{h}$ decreases with the increase of DTR. When DMT > 32.461°C, $b_{h}$ increases with the increase of DTR. Fig 6(D) shows that in Jinghong, when DTR = 0°C, $b_{h}$ increases with the increase of DMT; when DMT is 18–20°C, $b_{h}$ is almost unaffected by the change of DTR; when DMT < 18°C, $b_{h}$ increases with the increase of DTR; when DMT > 20°C, $b_{h}$ decreases with the increase of DTR.

In summary, due to the significant local temperature difference between Guangzhou and Jinghong, except a, the other three mosquito vector parameters (n, $b_{m}$, $b_{h}$) are also significantly different in terms of the degree of influence by temperature.

#### 3.3.3. Change trend of mosquito vector parameters.

**Fig 7:**
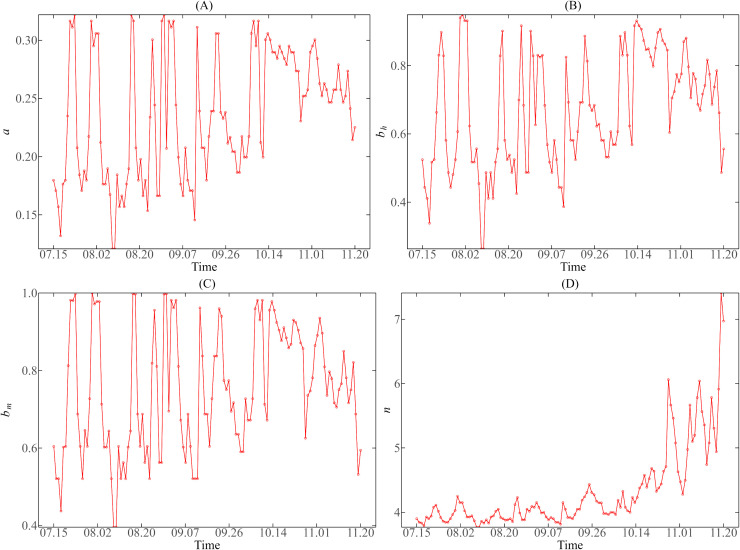
Variation trend of mosquito vector parameters in Guangzhou during the dengue fever epidemic period from July 15 to November 20, 2019. (A) Biting rate 𝐚; (B) The transmission probability from the vector to humans per bite ${𝐛}_{h}$; (C) The transmission probability from human to the vector per bite ${𝐛}_{\text{m}}$; (D) External incubation period 𝐧.

**Fig 8:**
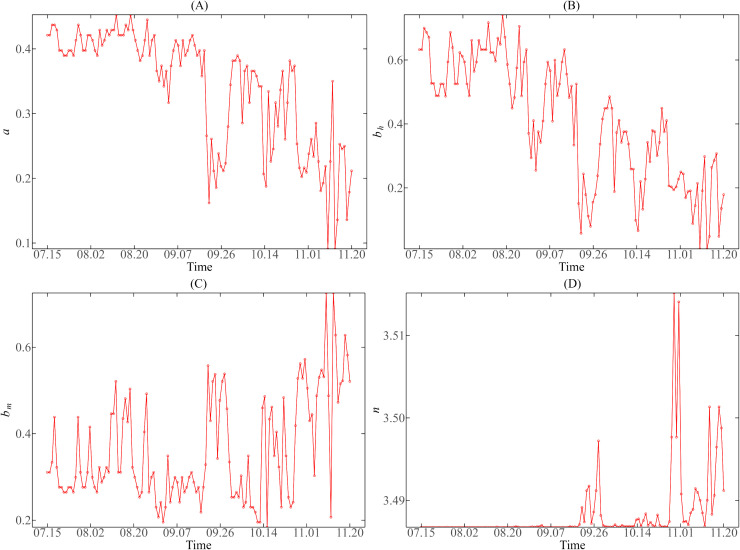
Variation trend of mosquito vector parameters in Jinghong during the dengue fever epidemic period from July 15 to November 20, 2019. (A) Biting rate 𝐚; (B) The transmission probability from the vector to humans per bite ${𝐛}_{h}$; (C) The transmission probability from human to the vector per bite ${𝐛}_{m}$; (D) External incubation period 𝐧.

We also presented the trend chart of vector parameter values in Guangzhou and Jinghong during July 15 to November 20, 2019 (as shown in Fig 7 and Fig 8) to facilitate comparative analysis.

From Fig 7 and Fig 8(A), there is a significant difference in the change trend of mosquito bite rate (a) between Guangzhou and Jinghong during the dengue fever epidemic in 2019. In contrast, the mosquito bite rate in Jinghong ranged from 0.09 to 0.45. In the early stage of the epidemic (July 15 to August 20), the change of DMT was small, and a changed around 0.4. In the later stage, the change of DMT increased. The fluctuation range of a also increased significantly but stabilized below 0.4 due to the decrease in temperature.

As shown in Fig 7(B), the $b_{h\;}$ in Guangzhou was affected by temperature change and fluctuated greatly, ranging from 0.26 to 0.95. In the later period of the epidemic, it basically remained between 0.5 and 0.9, with no obvious downward or upward trend overall. In contrast, Fig 8(B) shows that the $b_{h\;}$ of Jinghong varies from 0.01 to 0.74. Before September, the $b_{h\;}$ changes at about 0.6, and then drops to 0.4 after September, showing a downward trend.

It can be seen from (C) of Fig 7 and Fig 8 that during the dengue epidemic in 2019, the $b_{m}$ of Guangzhou varied between 0.4 and 1, while that of Jinghong fluctuated between 0.18 and 0.73. On the whole, the level of $b_{m}$ in Guangzhou is higher than that in Jinghong, and the fluctuation frequency of $b_{m}$ in Guangzhou is larger.

In addition, it can also be seen from Fig 7 and Fig 8 (D) that, from July to November, with the decrease of average temperature, n in Guangzhou and Jinghong showed an increasing trend. The external incubation period (n) of mosquito vectors in Guangzhou was about 4–7 days, and that of mosquito vectors in Jinghong was about 3–4 days. The incubation period was prolonged in Jinghong, but the actual variation of n was very limited due to the low DTR.

The four mosquito vector parameters in Guangzhou and Jinghong showed different changing trends during the dengue fever epidemic in 2019, which also reflected the influence of regional climate differences on mosquito vector behavior.

### 3.4. Sensitivity analysis

**Fig 9:**
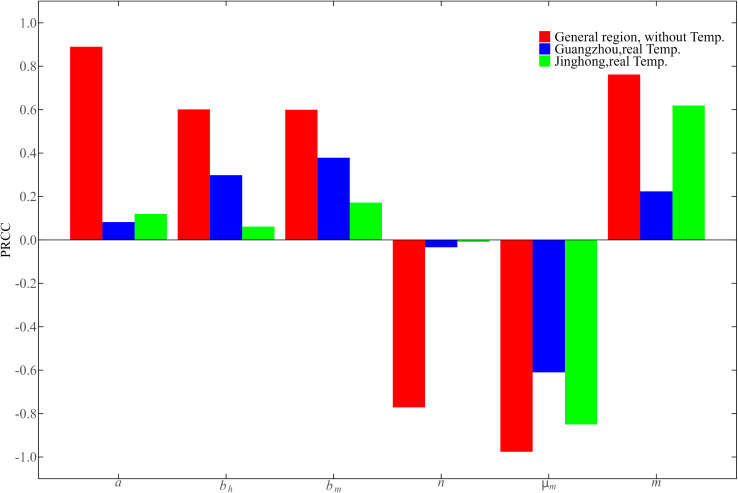
PRCC sensitivity analysis of six mosquito vector parameters $𝐚,\;b_{h},\;b_{m},\;\mu_{m},\;n\;\text{and}\;m$ to basic reproduction number ${𝐑}_{0}$. The red, blue and green bars are for the temperature without consideration, the temperature of Guangzhou and the temperature of Jinghong respectively.

To identify the contribution of the six key parameters ($a,\;b_{h},\;b_{m},\;\mu_{m},\;n\;\text{and}\;m$) on the basic reproduction number ${\;R}_{0},$ and to better compare the results of the sensitivity analysis, including comparisons within the same area regarding the presence or absence of temperature, as well as comparisons between different areas, we examined the impact of input parameters on dengue transmission through sensitivity analysis under two scenarios: without and with involved temperature effects (DMT and DTR). The sensitivity analysis results are shown in Fig 9.

For the sensitivity analysis without involved temperature, we directly give the range of vector parameters: the range of a is (0.15,0.55), the range of $b_{h}$ and $b_{m}$ is (0.3,0.8), the range of $\mu_{m}$ is (0.1,0.5), the range of n is (7,12), and the range of m is (3,15) [3,27,33–35]. As shown in the red bar chart in Fig 9, vector mortality ($\mu_{m}$) is the most sensitive parameter, which is negatively correlated with ${\;R}_{0}$ (PRCC= −0.9758), and the secondary sensitive parameter is mosquito bite rate (a), which is positively correlated with ${\;R}_{0}$ (PRCC= 0.88981). The external incubation period (n) of dengue virus was negatively correlated with ${\;R}_{0}$ (PRCC= −0.7715), $b_{h}$, $b_{m}$ and m were positively correlated with $R_{0}$, and the correlation was strong.

For the sensitivity analysis with involved temperature, let DMT, DTR, ${\;\mu }_{m}\;and\;m$ as input parameters, $a,\;b_{h}$, $b_{m}$, n are calculated by Eq. (2). According to the actual temperature of Guangzhou and Jinghong during the dengue fever epidemic in 2019, the variation ranges of DMT and DTR in Guangzhou were (19,34) and (3,12), and the variation ranges of DMT and DTR in Jinghong were (11,22) and (2,12), respectively. The value ranges of ${\;\mu }_{m}$ and m are the same as the preceding of the case without involved temperature. As can be seen from the blue and green bar charts in Fig 9, mosquito mortality $\mu_{m}$ is still the most sensitive in both regions and is negatively correlated with $R_{0}$ (Guangzhou, PRCC=−0.6100; Jing Hong, PRCC=−0.8502). At the same time, $a,\;b_{h}$, $b_{m}$ and m were positively correlated with $R_{0}$ in Guangzhou, among which $b_{m}$ was a mosquito vector parameter that was secondly sensitive to $R_{0}$ (PRCC=0.3785), a and n were weakly correlated with $R_{0}$. In Jinghong, $a,\;b_{h},\;b_{m}\;\text{and}\;n$ were weakly correlated with $R_{0}$ owing to their absolute values of PRCC were all less than 0.2. m was the second most sensitive mosquito vector parameter to $R_{0}$, and had a strong positive correlation with $R_{0}$ (PRCC=0.6190). According to Fig 9, the PRCC values considering temperature (see blue and green bars) in both two regions are lower overall than those without considering temperature (see red bars). This indicates that the sensitivity analysis without considering temperature might overestimate the contribution of the parameters to $R_{0}$. For regional differences, the contributions of $a,\mu_{m}\;\text{and}\;m\;$to $R_{0}$ in Jinghong are greater than those in Guangzhou. However, the conclusions for $b_{h}\;$and $b_{m}$ are opposite. In summary, the results of sensitivity analysis show that vector control is the most effective epidemic prevention measure in both Guangzhou and Jinghong, and temperature also affects the development of dengue fever epidemic to some extent.

### 3.5. Effects of temperature and imports on dengue fever epidemic

#### 3.5.1. Relationship between temperature and effective reproduction number.

**Fig 10:**
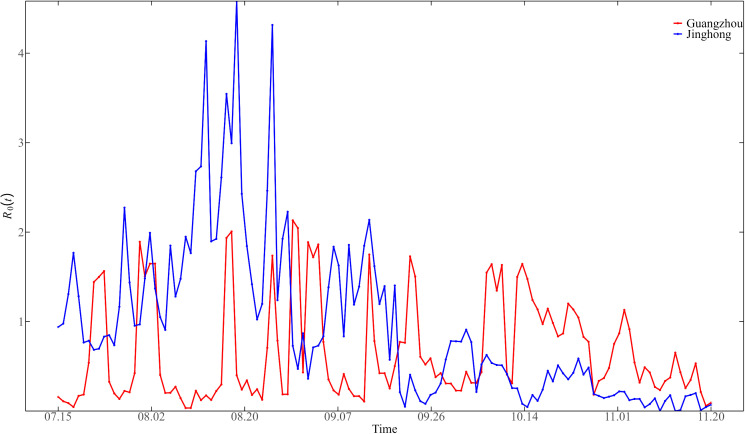
The effective reproduction number ${𝐑}_{\text{0}}(t)$ in Guangzhou (red line) and Jinghong (blue) from July 15 to November 20, 2019.

According to the results of relevant analysis, mosquitoes, as the vector of dengue virus, have a significant impact on the dengue epidemic. Therefore, we considered a comparative analysis of the effective reproduction number $R_{0}(t)$ in Guangzhou and Jinghong during the dengue epidemic in 2019 (as shown in Fig 10) to explore the impact of mosquitoes on dengue cases.

As can be seen from the figure, the effective reproduction number of Guangzhou fluctuates between 0 and 2. The effective reproduction number of Jinghong fluctuated between 0 and 4.3. In the early stage of the epidemic, the effective reproduction number of Jinghong was significantly higher than that of Guangzhou. The effective reproduction number of Jinghong reached its peak in mid-late August, and the peak value exceeded 4, while after the end of September, the effective reproduction number of Jinghong stabilized below the level of 1. Although the effective reproduction number in Guangzhou showed a multi-peak trend, there was no obvious upward or downward trend overall, and it remained below the level of 2. Considering that since the outbreak of the epidemic in 2014, the Guangzhou Municipal Government has actively taken epidemic prevention measures and popularized epidemic prevention knowledge every year, manual prevention and control measures have played an effective role.

Since March 2019, Guangdong has begun to deploy epidemic prevention measures, carrying out mosquito surveillance sites in various cities, launching a special campaign for dengue prevention and control, popularizing dengue prevention knowledge, calling on the public to pay attention to hygiene measures and other measures to reduce the number of mosquitoes. Therefore, it can be seen from Fig 10 that during the epidemic period, the overall effective reproduction number of Guangzhou remained at a low level. From the end of September to the end of October, $R_{0}(t)$ fluctuated frequently due to the increase in the number of people in Guangzhou during holidays and rainy weather. After the end of October, affected by the overall temperature drop in Guangzhou, the continuous mosquito control and epidemic prevention actions in the whole province, $R_{0}(t)$ was generally stable below 1.

In early March 2019, Yunnan Province cooperated with many neighboring Southeast Asian countries to strengthen cross-border joint prevention and control of dengue fever. Along the border, it deployed surveillance stations to monitor mosquito vector data weekly. These efforts have formed a strong defense line against insect-borne diseases. Therefore, it can be seen from Fig 10 that from mid-July to mid-August in the early stage of the epidemic, $R_{0}(t)$ in Jinghong was controlled near the level of 2. The peak period of dengue fever in Jinghong was from July to September, during which the temperature was high and rainy, which was suitable for mosquito breeding. Affected by the climate, the effective reproduction number in Jinghong showed a sharp increase trend after mid-August and reached the peak of the entire epidemic period near August 20, with $R_{0}(t)$ exceeding 4. From the end of September, the temperature began to drop (the daily average temperature began to drop below 20 °C), and $R_{0}(t)$ also began to drop and stabilized below 1 in the later period.

#### 3.5.2. Effect of imports on dengue fever epidemic.

From Fig 10, we can see that during the period from July 15 to November 20, 2019, although the effective reproduction number $R_{0}(t)$ in Guangzhou was not high, there were still many dengue fever cases reported, and the number of new dengue fever cases in Jinghong in 2019 reached the highest of hundreds. According to the relevant reports of the epidemic in the two places at that time, the dengue fever epidemic in Guangzhou and Jinghong in 2019 was also affected by imported cases from abroad. Note that our available data doesn’t distinguish between local and imported cases. In order to study the effect of imported cases on dengue fever epidemic, we use the imported case coefficient (imp) to quantify the intensity of case importation in the dynamic model (See S1). Although we do not explicitly track the absolute count of imported cases, imp directly scales the force of infection from imported cases to mosquitoes. Elevated imp values indicate a higher number of imported cases, which serve as sources of dengue virus introduction into the local transmission cycle via mosquito-borne spread. Consequently, imp exerts a significant influence on the number of daily new cases. Therefore, by changing the import coefficient imp of the two places in the dynamic model, we can explore the impact of imported cases on the dengue fever epidemic in Guangzhou and Jinghong in 2019.

**Fig 11:**
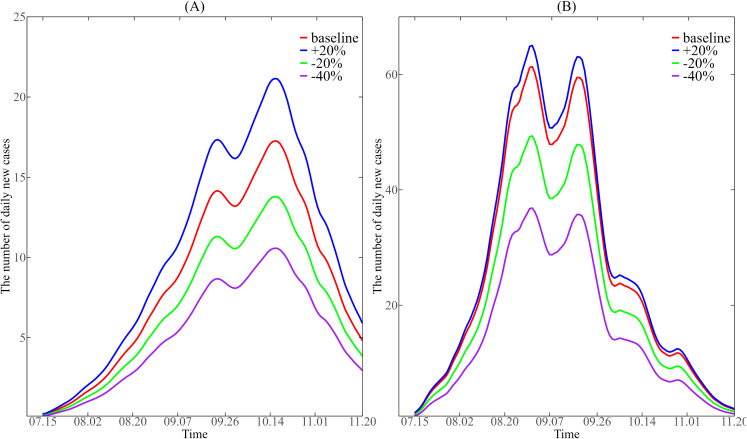
Impact of different import coefficients (𝐢𝐦𝐩) on the daily number of new cases in Guangzhou (A) and Jinghong (B) from July 15 to November 20, 2019. The baseline values of $\begin{array}{c} imp \end{array}$ for Guangzhou (3.7542) and for Jinghong (191.5245) are from the result of MCMC parameter estimation*.* The baseline values are increases by 20%, decreases by 20%, and decreases by 40% respectively. Substitute $\begin{array}{c} imp \end{array}$ back into the dynamic model to simulate the corresponding daily number of new cases.

As shown in Fig 11, we adjusted the estimated input coefficient imp (red line) in the dynamic models of Guangzhou and Jinghong. The baseline values of imp were estimated via MCMC parameter estimation. The estimated baseline values for imp were 3.7542 in Guangzhou and 191.5245 in Jinghong (see S1). The values of imp for Jinghong is much higher than that of Guangzhou. It indicates that Jinghong is more substantially affected by cross-border transmission. Therefore, Jinghong was more affected by imported cases from abroad, which may be related to the geographical characteristics of its adjacent border. Specifically, we increased imp by 20% (blue line), decreased imp by 20% (green line), and decreased imp by 40% (purple line), respectively, and brought these adjustments back into the model to simulate the number of new dengue cases per day under different scenarios. It can be observed from Fig 11(A) that when the imp of Guangzhou is increased or decreased by 20% on the basis of the original imp, the daily number of new cases will increase or decrease by 5 cases at most, and with each reduction by 20%, the reduction range of the corresponding number of cases tends to stabilize. Comparatively, Fig 11(B) shows that when the original imp of Jinghong is reduced by 20%, the daily number of new cases is reduced by 10 cases at most, and when it is increased by 20%, the daily number of new cases is increased by 5 cases at most. When the import coefficient imp increased by 20% in both cities, Jinghong showed a significantly smaller relative increase in the number of dengue cases compared to Guangzhou. This suggests that the number of dengue cases in Jinghong was already closer to a saturation level influenced by imported cases. From the perspective of epidemic prevention and control, strengthening the management of overseas population imports can help reduce the import coefficient imp, thereby lowering the number of dengue cases to some extent.

## 4. Discussion and conclusion

This paper aims to study the impact of temperature and imported cases on the epidemic situation of dengue fever. Therefore, Guangzhou and Jinghong with large differences in temperature and imported cases were selected as research objects. The daily new cases and temperature data of the two cities in 2019 were collected. This article has the following three main highlights. Firstly, compared with a study of the same type using statistical model to study the impact of temperature on the dengue fever epidemic in Guangzhou in 2014 [8], this paper focuses on the use of statistical model and dynamic model to study the influence of temperature and imported cases on the epidemic of dengue fever. Secondly, given that mosquito vector surveillance data are often incomplete, this paper provides a solution for missing data with the help of epidemiological dynamics models. Finally, most of the previous studies focused on a certain region. In this paper, Guangzhou and Jinghong, two cities with large differences between temperature and imports, were selected as typical cases to compare the effects of temperature and imports on the dengue fever epidemic in 2019.

To assess the impact of the number of mosquito vectors on the dengue outbreak, we collected daily data on the number of new cases and temperature from 15 July to 20 November 2019 in the two places and used the dengue epidemic dynamics model to estimate the mosquito vector related data during the outbreak. Pearson correlation analysis showed that it was reasonable to use the model to obtain mosquito data. In addition, cross-correlation analysis showed that there was a feedback and contemporaneous relationship between mosquito numbers and the number of new cases. For Guangzhou and Jinghong, the lag time of the interaction between the mosquito population and the number of new cases per day was different, the interaction lasted longer in Jinghong. This means that the temporal characteristics of mosquito effects on disease transmission are different in the two regions, which may be related to local ecological environment, climatic conditions and disease transmission patterns.

To investigate the effect of temperature on mosquito vector parameters, we established the relationship between temperature (DMT and DTR), mosquito vector parameters and reproduction number, and estimated the mosquito vector parameters in Guangzhou and Jinghong using the maximum likelihood estimation (MLE) method. Contour maps were used to study the DMT and DTR effects on vector parameters. The mosquito vector parameters were affected by temperature in a complex way. Due to the different DMT and DTR in Guangzhou and Jinghong, the influence of temperature patterns on the mosquito vector parameters was also different. Although the bite rate (a) was only linearly correlated with DMT in both regions, the a in Jinghong was slightly higher than that in Guangzhou. At the same time, in the parts where DTR and DMT overlap, the change pattern of the probability of transmission from mosquito to human per bite ($b_{h}$) is similar. The probability of mosquito-borne transmission ($b_{m}$) per bite was significantly different in Guangzhou and Jinghong under different temperature conditions. The results showed that the values, variation ranges and fluctuation frequencies of the four mosquito vector parameters ($a,b_{h},b_{m},n$) were significantly different in the two regions. The largest difference between the two regions was the external incubation period (n). The external incubation period n in Jinghong was significantly shorter than that in Guangzhou, which was more conducive to the transmission of dengue virus. In addition, the effects of six vector parameters ($a,b_{h},b_{m},\mu_{m},n,m$) on the basic reproduction number in the two areas were studied by sensitivity analysis. The most sensitive parameters to the basic reproduction number were the mosquito mortality rate and the virus transmission rate between mosquito vectors and humans. The results of the sensitivity analysis demonstrated that DMT and DTR played a key role in the development of the dengue outbreak in 2019 and the spread of the virus.

Effective reproduction number $R_{\text{0}}(t)$ is an important indicator of the development of the epidemic over time [37,38]. Analyzing the change trend of $R_{\text{0}}(t)$ is helpful to evaluate the effectiveness of the implementation of prevention and control measures. Since 2019, the incidence of dengue fever in Southeast Asia and other regions has increased compared with previous years and imported dengue cases have appeared in Guangdong and Yunnan provinces. As a result, both Guangdong and Yunnan provinces have begun to deploy epidemic prevention measures ahead of the dengue fever epidemic. For example, on March 15, Guangzhou launched a special campaign to prevent dengue fever, including popularizing knowledge of dengue prevention and calling on the public to pay attention to hygiene to reduce the number of mosquitoes. In the same period, Jinghong also carried out joint prevention and control work with neighboring countries. Fig 10 shows that $R_{\text{0}}(t)$ in Guangzhou and Jinghong remained at a low level before July 15 to August 10. In particular, $R_{\text{0}}(t)$ in Guangzhou remained below 2 during the entire epidemic phase, with no obvious peak, which indicates the effectiveness of continuous mosquito control and epidemic prevention actions. Meanwhile, Guangzhou has more experience and more effective execution in dengue epidemic supervision, disease prevention and control, and public health awareness popularization.

Studies have demonstrated that dengue is an imported disease in China [39]. Therefore, we also adjust the input coefficient parameter imp in the dynamic models of Guangzhou and Jinghong respectively for comparative analysis. We found that Jinghong was more affected by imported cases, which may be related to its geographical characteristics adjacent to the border. Frequent population movements between China and foreign countries made imported cases an important factor affecting the local dengue epidemic. However, when imp is reduced, the daily number of new cases in Guangzhou and Jinghong will decrease significantly, which indicates that the epidemic prevention measures to control the management of imported cases from abroad will be effective in the prevention and control of dengue epidemic and will be more effective in Jinghong.

Several papers have studied the factors affecting the transmission of dengue fever, and the dynamic model has been widely applied and provides a clear framework for the study of the transmission of mosquito-borne diseases [2,16]. A new SEI-SEIR model has been proposed to better reveal the transmission mode of dengue fever [16]. This paper, based on this model, combined temperature and case data to solve the evaluation problem of unavailable authentic mosquito vector surveillance data, and verified the feasibility of this scheme by correlation analysis. Meanwhile, the influence of temperature on dengue fever epidemic has been studied in the past using statistical analysis [3,8]. However, these studies have generally focused on a particular region and assessed the impact of certain factors on dengue transmission without delving into how these patterns of impact might change under different conditions. Therefore, we need to conduct a comparative analysis of different outbreak cases to explore the factors that contribute to this difference. For example, by changing different climate and input parameters in the dynamic model to create different scenarios, the main causes of different outbreak patterns of dengue fever in Guangzhou in 2013 and 2014 were analyzed [9]. Different from this longitudinal comparison study, this paper selects two regions with significant differences in temperature and geographical location in the same period, namely Jinghong and Guangzhou, for horizontal comparison. Since the two regions have different climatic and geographical conditions, we can explore the pattern of temperature and imported cases on dengue epidemics through comparative studies.

The results of this study show that the difference in temperature, imported cases and epidemic prevention measures are all important factors leading to the difference in dengue epidemic prevalence between Guangzhou and Jinghong in 2019. Therefore, in the future prevention and control of dengue disease, it is necessary not only to pay attention to weather conditions and adopt human intervention measures, but also to adapt to local conditions. For areas that are heavily affected by overseas population movements, strengthening the management of overseas population importation may be more effective in reducing the local transmission of dengue virus.

In fact, there are obvious differences between Guangzhou and Jinghong in many aspects, such as socioeconomic, population density and population mobility, but this paper does not consider whether these differences may have an impact on the transmission of dengue virus in different regions, which will require further research. Notwithstanding these limitations, our generalized modeling framework achieved consistent accuracy with observed transmission patterns in both regions through localized calibration. However, our model does not account for species-specific behaviors. Future studies could first incorporate ecological differences between local vectors (e.g., indoor preference of Aedes aegypti and outdoor activity characteristics of Aedes albopictus). Furthermore, our primary focus was on estimating the potential Aedes population near human settlements in urban and rural areas, where mosquito breeding predominantly occurs in artificial water containers, such as reservoirs, flowerpot and so on, rather than precipitation-dependent habitats. Although the current study focused exclusively on temperature effects, the ecological activities of Aedes albopictus and Aedes aegypti are jointly affected by temperature and rainfall, and the influence mechanisms are different [40,41]. Our future studies will incorporate both factors for more comprehensive modeling formulation.

## Supporting information

S1 File Dengue epidemic model and parameter estimation. (DOCX)

S2 File Relevant data mentioned in subsection 2.2. (XLSX)
