# Supplementary material for: Impact of temperature and imported cases on the spread and control of dengue fever: Case study of 2019 dengue fever epidemic in Guangzhou and Jinghong cities, China
Source: PLoS Negl Trop Dis. 2025 Sep 22;19(9):e0013472. doi: 10.1371/journal.pntd.0013472 (PMC12453222; doi:10.1371/journal.pntd.0013472)
Supplement: S1 File — (DOCX) [file pntd.0013472.s001.docx]

**S1：Dengue epidemic model and parameter estimation**

SEIR model is a commonly used infectious disease dynamic model, which is mainly used to describe the transmission process of infectious disease in the population. In this paper, based on the coupled human-mosquito transmission mechanism of dengue fever, and further extended on the basis of the SEIR model and other related studies [1,2], a dengue fever transmission model (*ELPSE-SEIR*) was established [3]. The number of larvae and the number of adult mosquitoes estimated by this model during the aquatic period will be used as mosquito vector data in this study.

Description of mosquito vector dynamics: The development process of Aedes mosquitoes goes through egg stage ($E$), larval stage ($L$), pupa stage ($P$), and then goes through emergence into adult Aedes mosquito. After biting dengue patients, susceptible adult Aedes mosquitoes ($S_{m}$) transform into latent species ($E_{m}$) with a certain probability, and then after a period of incubation, they transform into infected species ($I_{m}$) with a certain probability. Description of human population dynamics: Susceptible people ($S_{h}$) are bitten by infected Aedes mosquitoes with a certain probability to enter the incubation period ($E_{h}$), and then after a period of incubation, with a certain probability to transform into infected people ($I_{h}$), after treatment, they become recovered ($R_{h}$).

The dynamic flow chart of the coupled transmission of dengue fever between humans and mosquitoes is shown in **Fig 1**, and the corresponding dynamical system is shown in Eq. (1). Based on the dynamic model, combined with the daily number of new dengue cases and climate data in Guangzhou and Jinghong in 2019, we estimated the mosquito vector data during the dengue epidemic in these two cities, including the number of mosquitoes in the aquatic stage and the number of adult mosquitoes. To reduce the number of unknow parameters involved in the model, the values of several parameters were firstly obtained by referring to relevant literatures. For the estimation of unknown parameters, we use *Markov Chain Monte Carlo* (*MCMC*) algorithm [4], perform 20000 simulations and 10000 annealing algorithms for different parameter combinations. The meanings of parameter results, expressions and initial values related to the model are shown in **Table 1**, **Table 2** and **Table 3**.

$$S_{m}$$

$$E_{m}$$

$$I_{m}$$

$$S_{h}$$

$$E_{h}$$

$$I_{h}$$

$$R_{h}$$

$$\phi h$$

$$f_{e}$$

$$f_{l}$$

$${\sigma f}_{p}$$

$$b_{hv}$$

ε

$$\mu_{e}$$

$$\mu_{l}$$

$$\mu_{p}$$

$$\mu_{m}$$

$$\mu_{m}$$

$$\mu_{m}$$

$${AN}_{h}$$

$$b_{vh}$$

δ

γ

$$d$$

$$d$$

$$d$$

$$d$$

**Fig 1.** Flow diagram on the dynamical transmission of dengue virus among mosquitoes and humans.

Based on the flow diagram in **Fig 1**, the corresponding dengue epidemic model is given as follows:

$$\left\{ \begin{aligned} \frac{dE}{dt} = \phi h\left( S_{m}+E_{m}+I_{m} \right)-\left( f_{e}+\mu_{e} \right)E, \\ \frac{dL}{dt} =f_{e}E-\left( f_{l}+\mu_{l}(1+L/k_{l}) \right)L, \\ \frac{dP}{dt} =f_{l}L-\left( f_{p}+\mu_{p} \right)P, \\ \frac{dS_{m}}{dt}= \sigma f_{p}Pe^{-\mu_{em}\left( 1+\frac{P}{k_{p}} \right)}-b_{hv}S_{m}\frac{I_{h}+imp}{N_{h}}-\mu_{m}S_{m} , \\ \frac{dE_{m}}{dt} =b_{hv}S_{m}\frac{I_{h}+imp}{N_{h}}-\varepsilon E_{m}-\mu_{m}E_{m}, \\ \frac{dI_{m}}{dt}=\varepsilon E_{m}-\mu_{m}I_{m}, \\ \frac{dS_{h}}{dt}=AN_{h}-b_{vh}S_{h}\frac{I_{m}}{N_{h}}-dS_{h}, \\ \frac{dE_{h}}{dt}=b_{vh}S_{h}\frac{I_{m}}{N_{h}}-\delta E_{h}-dE_{h}, \\ \frac{dI_{h}}{dt}=\delta E_{h}-\gamma I_{h}-dI_{h}, \\ \frac{dR_{h}}{dt}=\gamma I_{h}-dR_{h}. \end{aligned} \right.(1)$$

Denote $M(t)$as the number of adult mosquitoes on day $t.$ From model (1), we can obtain $M(t){=S}_{m}\left( t \right)+E_{m}\left( t \right)+I_{m}(t)$.

**Table 1** The initial value and state variables of dengue dynamic model.

| Parameter | Definition | Initial value | Reference |
| --- | --- | --- | --- |
| $E$ | Number of Aedes mosquito eggs | $N_{h}\times K$ | [5] |
| $L, P$ | Number of larvae,  pupae | 0 | — |
| $S_{m}$*,* $E_{m}$*,* $I_{m}$ | Number of susceptible, latent, infected adult mosquitoes | 0 | — |
| $S_{h}$ | Number of susceptible  people | $N_{h}$ | [6,7] |
| $E_{h}$, $I_{h}$, $R_{h}$ | Number of incubated, infected, recovered people | 0 | — |
| $N_{h}$ | Number of permanent  people | — | [6,7] |

[Note] $N_{h}$ of Guangzhou is 15305900 and that of Jinghong is 642737. These two figures come from the 2019 statistical Yearbooks of both places

**Table 2** The parameters of dengue dynamic model (1).

| Parameter | Definition | Expression (Value) | Reference |
| --- | --- | --- | --- |
| $\mu_{e}$ | Mortality rate of mosquito  egg | 0.1 | [8] |
| $\mu_{em}$ | Mortality rate at emergence stage | 0.1 | [9] |
| $imp$ | Dengue case import  coefficient | — | Parameter estimation |
| $\phi$ | Egg-laying rate of adult female mosquitoes | (2) | [10] |
| $h$ | The survival rate of eggs |  | Parameter estimation |
| $f_{e}$ | Egg hatching rate | (3) | [11] |
| $f_{l}$ | Larval development rate | (4) | [11] |
| $f_{p}$ | Pupa development rate | (5) | [11] |
| $\mu_{l}$ | Larval death rate | (6) | [11] |
| $\mu_{p}$ | Pupa death rate | (7) | [11] |
| $\mu_{m}$ | Death rate of adult  mosquitoes | (8) | [10] |
| $a$ | Biting rate | (9) | [12] |
| $b$ | Infection probability per bite (mosquito → human) | (10) | [13] |
| $c$ | Infection probability per bite (human → mosquito) | (11) | [13] |
| $b_{v}$ | Effective transmission coefficient (human → mosquito) | 1 | [14] |
| $b_{h}$ | Effective transmission coefficient (mosquito → human) | — | Parameter estimation |
| $b_{hv}$ | Effective transmission rate per bite (human → mosquito) | $b_{hv}=acb_{v}$ | [14] |
| $b_{vh}$ | Effective transmission rate per bite (mosquito→ human) | $b_{vh}=abb_{h}$ | [14] |
| $A$ | Natural birth rate | — | — |
| $d$ | Natural mortality | — | — |
| $\sigma$ | Proportion of female Aedes mosquitoes | 0.5 | [10] |
| $\delta$ | Rate of latency | 1/5 | [15] |
| $\gamma$ | Rate of recovery | 1/6 | [15] |
| $k_{l}, k_{p}$ | Environmental capacity of larva and pupa | $N_{h}\times3$ | [5] |

[Note] $K$ is the initial ratio of the number of eggs to the population. The value of $K$ is assumed to be 3 in both two places.

$$\phi=-5.4+1.8T-0.2124T^{2}+0.01015T^{3}-0.0001515T^{4}, (2)$$

$$f_{e}=0.5070\exp\left[ -\left( \frac{T-30.85}{12.82} \right)^{2} \right], (3)$$

$$f_{l}=0.1727\exp\left[ -\left( \frac{T-28.40}{10.20} \right)^{2} \right], (4)$$

$$f_{p}=0.6020\exp\left[ -\left( \frac{T-34.29}{15.07} \right)^{2} \right], (5)$$

$$\mu_{l}=\min\left\{ 1,\frac{1}{\left| -0.130 5T^{2}+3.868T+30.83 \right|} \right\}, (6)$$

$$\mu_{p}=\min\left\{ 1,\frac{1}{\left| -0.150 2T^{2}+5.057T+3.517 \right|} \right\}, (7)$$

$$\mu_{m}=0.869 2-0.159T+0.011 16T^{2}-3.408\times{10}^{-4}T^{3}+3.809\times{10}^{-6}T^{4}, (8)$$

$$a=2.02\times{10}^{-4}T\left( T-13.35 \right)\left( 40.08-T \right)^{\frac{1}{2}}, (9)$$

$$b=\left\{ \begin{aligned} 0.072 9T-0.903 7, 12.4 ℃<T<26.1 ℃ \\ 1, 26.1 ℃<T<32.5 ℃ \\ 0, others \end{aligned} \right. (10)$$

$$c=\max\left\{ 1.044\times{10}^{-3}T\left( -12.286 \right)\left( 32.461-T \right)^{\frac{1}{2}}, 0 \right\}. (11)$$

**Table 3** Results of the estimated parameters of model (1) for the two regions as well as the population numbers $N_{h}$.

| District | $b_{h}$ | $imp$ | $h$ | $A$ | $d$ |
| --- | --- | --- | --- | --- | --- |
| Guangzhou | 0.002846 | 3.7542 | 0.993536 | 0.00040712 | 0.00014082 |
| Jinghong | 0.031380 | 191.5245 | 0.768680 | 0.00029808 | 0.00013699 |

**Reference**

1. Zhu G, Liu T, Xiao J, Zhang B, Song T, Zhang Y, et al. Effects of human mobility, temperature and mosquito control on the spatiotemporal transmission of dengue. Sci Total Environ. 2019; 651: 969–978. doi: 10.1016/j.scitotenv.2018.09.182

2. Jia P, Lu L, Chen X, Chen J, Guo L, Yu X, et al. A climate-driven mechanistic population model of Aedes albopictus with diapause. Parasites Vectors. 2016; 9: 175. doi: 10.1186/s13071-016-1448-y

3. Zhao J, He G, Xiao J, Zhu G, Liu T, Hu J, et al. The mechanism of temperature affecting the transmission of dengue fever and the effect of future temperature change on its transmission risk. J Occup Environ Med. 2022; 39: 309–314.

4. Shi B, Liu J, Zhou XN, Yang GJ. Inferring plasmodium vivax transmission networks from tempo-spatial surveillance data. PLoS Negl Trop Dis. 2014; 8: e2682. doi: 10.1371/journal.pntd.0002682

5. Liao CM, Huang TL, Cheng YH, Chen WY, Hsieh NH, Chen SC, et al. Assessing dengue infection risk in the southern region of Taiwan: implications for control. Epidemiol Infect. 2015; 143: 1059–1072. doi: 10.1017/S0950268814001745

6. Guangzhou Statistical Yearbook. Beijing: China StatisticsPress; 2020.

7. Jinghong Statistical Yearbook. Beijing: China StatisticsPress; 2020.

8. Liu-Helmersson J, Rocklöv J, Sewe M, Brännström Å. Climate change may enable *Aedes aegypti* infestation in major European cities by 2100. EnvironRes. 2019; 172: 693–699. doi: 10.1016/j.envres.2019.02.026

9. Cailly P, Tran A, Balenghien T, L’Ambert G, Toty C, Ezanno P. A climate-driven abundance model to assess mosquito control strategies. EcolModell. 2012; 227: 7–17. doi: 10.1016/j.ecolmodel.2011.10.027

10. Yang HM, Macoris MLG, Galvani KC, Andrighetti MTM, Wanderley DMV. Assessing the effects of temperature on dengue transmission. Epidemiol Infect. 2009; 137: 1179–1187. doi: 10.1017/S0950268809002052

11. Jia P, Chen X, Chen J, Lu L, Liu Q, Tan X. How does the dengue vector mosquito Aedes albopictus respond to global warming? Parasites Vectors. 2017; 10: 140. doi: 10.1186/s13071-017-2071-2

12. Mordecai EA, Cohen JM, Evans MV, Gudapati P, Johnson LR, Lippi CA, et al. Detecting the impact of temperature on transmission of Zika, dengue, and chikungunya using mechanistic models. PLoS Negl Trop Dis. 2017; 11: e0005568. doi: 10.1371/journal.pntd.0005568

13. Wesolowski A, Qureshi T, Boni MF, Sundsøy PR, Johansson MA, Rasheed SB, et al. Impact of human mobility on the emergence of dengue epidemics in Pakistan. Proc Natl Acad Sci USA. 2015; 112: 11887–11892. doi: 10.1073/pnas.1504964112

14. Lee H, Kim JE, Lee S, Lee CH. Potential effects of climate change on dengue transmission dynamics in Korea. PLoS One. 2018; 13: e0199205. doi: 10.1371/journal.pone.0199205

15. Wearing HJ, Rohani P. Ecological and immunological determinants of dengue epidemics. Proc Natl Acad Sci USA. 2006; 103: 11802–11807. doi: 10.1073/pnas.0602960103
